# Particle-based, Pfs230 and Pfs25 immunization is effective, but not improved by duplexing at fixed total antigen dose

**DOI:** 10.1186/s12936-020-03368-5

**Published:** 2020-08-28

**Authors:** Wei-Chiao Huang, Bingbing Deng, Moustafa T. Mabrouk, Amal Seffouh, Joaquin Ortega, Carole Long, Kazutoyo Miura, Yimin Wu, Jonathan F. Lovell

**Affiliations:** 1 Department of Biomedical Engineering, University at Buffalo, State University of New York, Buffalo, NY 14260 USA; 2 Laboratory of Malaria and Vector Research, National Institute of Allergy and Infectious Diseases, National Institutes of Health, Rockville, MD 20852 USA; 3 Department of Anatomy and Cell Biology, McGill University Montreal, Quebec, H3A 0C7 Canada; 4 PATH’s Malaria Vaccine Initiative (MVI), Washington, DC 20001 USA

**Keywords:** Pfs25, Pfs230, Malaria, Transmission-blocking vaccine, Liposomes

## Abstract

**Background:**

The *Plasmodium falciparum* sexual-stage surface proteins Pfs25 and Pfs230 are antigen candidates for a malaria transmission-blocking vaccine (TBV), and have been widely investigated as such. It is not clear whether simultaneously presenting these two antigens in a particulate vaccine would enhance the transmission reducing activity (TRA) of induced antibodies. To assess this, immunization was carried out with liposomes containing synthetic lipid adjuvant monophosphoryl lipid A (MPLA), and cobalt-porphyrin-phospholipid (CoPoP), which rapidly converts recombinant, his-tagged antigens into particles.

**Methods:**

His-tagged, recombinant Pfs25 and Pfs230C1 were mixed with CoPoP liposomes to form a bivalent vaccine. Antigens were fluorescently labelled to infer duplex particleization serum-stability and binding kinetics using fluorescence resonance energy transfer. Mice and rabbits were immunized with individual or duplexed particleized Pfs25 and Pfs230C1, at fixed total antigen doses. The resulting antibody responses were assessed for magnitude and TRA.

**Results:**

Pfs230C1 and Pfs25 rapidly bound CoPoP liposomes to form a serum-stable, bivalent particle vaccine. In mice, immunization with 5 ng of total antigen (individual antigen or duplexed) elicited functional antibodies against Pfs25 and Pfs230. Compared to immunization with the individual antigen, Pfs25 antibody production was moderately lower for the bivalent CoPoP vaccine, whereas Pfs230C1 antibody production was not impacted. All antibodies demonstrated at least 92% inhibition in oocyst density at 750 μg/mL purified mouse IgG in the standard membrane feeding assay (SMFA). At lower IgG concentrations, the bivalent vaccine did not improve TRA; antibodies induced by particleized Pfs25 alone showed stronger function in these conditions. In rabbits, immunization with a 20 µg total antigen dose with the duplexed antigens yielded similar antibody production against Pfs25 and Pfs230 compared to immunization with a 20 µg dose of individual antigens. However, no enhanced TRA was observed with duplexing.

**Conclusions:**

Pfs25, Pfs230 or the duplexed combination can readily be prepared as particulate vaccines by mixing CoPoP liposomes with soluble, recombinant antigens. This approach induces potent transmission-reducing antibodies following immunization in mice and rabbits. Immunization with bivalent, particleized, Pfs230 and Pfs25 did not yield antibodies with superior TRA compared to immunization with particleized Pfs25 as a single antigen. Altogether, duplexing antigens is straightforward and effective using CoPoP liposomes, but is likely to be more useful for targeting distinct parasite life stages.

## Background

Malaria was responsible for nearly half a million deaths in 2017 and is caused by *Plasmodium* parasites which are spread by female *Anopheles* mosquitoes [1]. The complex life cycle of *Plasmodium* parasites makes it difficult to develop an effective malaria vaccine. The first stage of the cycle is the pre-erythrocytic stage in which infected mosquitoes transfer parasites to a human host, where they invade the liver. After several cycles of replication, parasites migrate out of hepatocytes and enter the second stage, the blood stage, where they infect and reproduce in red blood cells in the human host. The third stage is the mosquito stage, in which parasites are taken up by a mosquito and undergo sexual reproduction in the mosquito gut. The pre-erythrocytic stage vaccine, RTS,S/AS01, which targets the circumsporozoite protein (CSP) protein of the parasite, is the most advanced malaria vaccine and prevented approximately 37% of cases in young African children, over 4 years and following 4 immunization doses [2]. Therefore, opportunities for improvements exist, which include developing vaccines targeting other life cycle stages. Vaccine approaches have been investigated in preclinical studies that target CSP along with other antigens including Pfs25 [3]. Transmission-blocking vaccines (TBV) induce immunity that targets the mosquito stage to block parasite transmission from mosquito to human host. TBVs are part of the World Health Organization’s *Malaria Vaccine Technology Roadmap,* but have yet to be assessed in large human clinical trials.

Several promising TBV antigen candidates have been identified for vaccine development, including Pfs25, Pfs230 and Pfs48/45 [4]. Pfs25 is a well-studied TBV antigen [5–7], which is only expressed in mosquito hosts [8]. The 25 kDa protein contains 11 disulfide bonds, therefore, it is important to produce properly folded Pfs25 in order to expose the correct epitopes [9–12]. Pfs25 has been studied in several clinical trials, but studies with Alum as an adjuvant or with Pfs25 in a virus-like particle format resulted in weak production of antibodies; and another clinical trial used Montanide ISA-51 as an adjuvant resulted in unacceptable local reactogenicity [13–15]. Pfs230 is another well-studied antigen that is expressed on gametocytes within human red blood cells before they are ingested by mosquitoes. The first cysteine motif domain (amino acids 587–730) of Pfs230 can induce antibodies with transmission-reducing activity (TRA) [16]. Pfs230C1 is a recombinant his-tagged N-terminal fragment of Pfs230 (amino acid 443–731), which has been shown to induce high level of transmission-reducing antibodies in mice [17].

One potential approach to improve the potency of a TBV is to combine multiple transmission blocking antigens into a single vaccine. In theory, this could result in synergy in (1) enhancing the immunogenicity of the antigens, or alternatively (2) the antibodies could combine to provide better transmission-blocking by targeting different parasite sites, with varied spatiotemporal features. Immunization with pre-fertilization (i.e., Pfs230) and post-fertilization (i.e., Pfs25) antigens could potentially induce more potent antibodies than either of single-antigen vaccines (Pfs230 alone or Pfs25 alone) with a fixed amount of total antigen. These two antigens have both advanced to human trials and both have been recently demonstrated to be effectively adjuvanted with the particle-inducing liposome system used in this work, described below [18, 19]. The rationale for using a fixed total dose of antigen was to objectively determine whether the combination of two TBV antigens would be more effective than any individual one. Pragmatically, the goal would be to determine what is the most effective TBV approach on the basis of the amount of the total injected protein dose, regardless of the number of antigens used. Menon et al. [20] concluded that a dual Pfs25 and Pfs230 vaccine was not superior to a Pfs25 single antigen immunization strategy when using a chimpanzee adenovirus 63 (ChAd63) and modified Vaccinia Ankara (MVA) viral prime-boost regime in mice. However, results could vary depending on vaccine platforms. In this work, this line of investigation is extended to TBV antigen duplexing using a particle-based protein approach.

The use of particle-based immune platforms is of broad interest [21]. Approaches such as the SpyCatcher system have shown promise for assembling antigens into more immunogenic assemblies [22]. Cobalt porphyrin-phospholipid (CoPoP) inserts into lipid bilayers and enables stable binding with polyhistidine-tagged proteins and peptides [23]. In mice and rabbits, this approach is effective with Pfs25 and multiple antigens can simultaneously be loaded onto CoPoP liposomes and induce a balanced immune response [19]. Similarly, preclinical studies showed that Pfs230C1 also is effectively adjuvanted with CoPoP liposomes [18]. The CoPoP system enables simple testing of whether immunization with antigens in a format that is bivalent, or duplexed (terms used interchangeably with bivalent in this work), can enhance TRA of induced antibodies, since particleization is achieved by simple mixing antigens and liposome without any additional purification. The commonly used TBV antigens, Pfs25 and Pfs230C1 were employed in this study, particleized with CoPoP liposomes. Monophosphoryl lipid A (MPLA) was also included in the liposomes, as this TLR-4 agonist is a promising adjuvant that has been included as a lipid component in clinical-stage vaccines, including for malaria and human papilloma virus [24].

## Methods

### Materials

His-tagged Pfs230C1 was produced in a baculovirus system as previously reported [17], as was Pfs25 [25]. CoPoP was produced as previously described [19]. The following lipids were used: 1,2-dipalmitoyl-sn-glycero-3-phosphocholine (DPPC, Corden # LP-R4-057, cholesterol (PhytoChol, Wilshire Technologies), synthetic monophosphoryl lipid A Phosphorylated HexaAcyl Disaccharide (PHAD, Avanti Cat # 699800P) and Monophosphoryl Lipid A-504 (PHAD504, Avanti # 699810).

### Liposome preparation

Liposomes were prepared by ethanol injection and nitrogen-pressurized lipid extrusion as previously described [19]. Ethanol and phosphate buffered saline (PBS) were preheated at 60 °C. Lipids were dissolved in preheated ethanol for 10 min, followed by adding preheated PBS into the samples for another 10 min at 60 °C. Liposomes were then passed through 200, 100 and 80 nm stacked polycarbonate filters in a lipid extruder (Northern Lipids) with nitrogen pressure. After extrusion, liposomes were dialyzed against PBS to remove ethanol. Final liposome concentration was adjusting to 320 μg/mL CoPoP, and passed through a 0.2 μm, sterile filter and stored at 4 °C. The liposome formulation had a mass ratio of [DPPC:CHOL:PHAD:CoPoP] [4:2:1:1] or alternatively, a reduced MPLA content formulation using structurally-similar PHAD504 was used for certain studies as indicated with a ratio of [DPPC:CHOL:PHAD504:CoPoP] of [4:2:0.4:1]. Liposome size and polydispersity were determined by dynamic light scattering with a NanoBrook 90 plus PALS instrument after 200-fold dilution in PBS.

### Particleization characterization

Protein binding with Pfs25, Pfs230C1 or the duplex was carried out by incubating protein and liposomes with a 1:4 mass ratio of total protein: CoPoP. Following incubation, the sample was subjected to microcentrifugal filtration (PALL Cat # 29300) and protein in filtrate was assessed by micro BCA (Thermo Cat # 23235). To determine binding saturation, 150 μL of Pfs230C1 (80 μg/mL) was mixed with 150 μL of liposomes containing 320 μg/mL of CoPoP or PoP in PBS for 3 h at room temperature. Samples were placed in a 100 kDa cut-off microcentrifugal filtration tube pre-rinsed with PBS and centrifuged at 1200 g for 60 min. The flow-through was collected and analysed with micro BCA assay to detect the amount of unbound proteins. Pfs230C1 binding to CoPoP liposomes or PoP liposomes was determined by measuring the BCA absorbance at 562 nm following the manufacturer protocol, using the following formula:  % antigen binding = (1−OD_562_ filtered CoPoP/MPLA + antigen/OD562 filtered antigen) × 100%. OD_562_ is the absorption at 562 nm as measured by a microplate reader.

### Electrophoretic mobility shift assay (EMSA)

Pfs25, Pfs230C1 or the duplex was carried out by incubating protein and liposomes with a 1:4 mass ratio of total protein: CoPoP. For native PAGE, loading dye was prepared containing 50% glycerol, Tris–HCl (0.25 M, pH 6.8) and 0.25% bromophenol blue. Loading dye was combined with the incubated samples, including Pfs25, Pfs230C1 and the duplex, and loaded into the Novex 4–12% Tris–Glycine Gel (Invitrogen #XV04120PK20). Tris–Glycine Native buffer (Invitrogen # LC2672) was used to run the gels. The samples were subjected to electrophoresis at 200 mV for 35 min. The gel was stained with Coomassie blue staining buffer (0.1% Coomassie Brilliant Blue G-250, 50% methanol and 10% acetic acid) for 30 min and destained with destaining buffer (40% methanol, 10% of acetic acid in deionized water) with overnight shaking at room temperature.

### Ni–NTA competition test

To further check protein binding stability, Ni–NTA Magnetic Beads (ThermoFisher # 88831) were used to compete with pre-bound proteins to the liposomes (1:4 mass ratio of total protein: CoPoP). Sufficient beads were added to ensure full binding of the free proteins in the sample. The samples were incubated with the beads for 30 min before the supernatant and magnetic beads were separated and collected using a magnetic separator (ThermoFisher # 12321D). The beads were the resuspended in PBS. Denaturing reducing loading dye was then added to all samples (supernatant and beads) and heated near 100 °C for 10 min. The samples were then loaded into Novex 4–12% Bis–Tris acrylamide gel (Invitrogen # NP0321BOX) and subjected to PAGE and bands were visualized with Coomassie staining.

### Antigen labelling with fluorescent dyes

Pfs230C1 was labelled with DY-405-NHS-Ester (DY-405, Dyomics). Labelling was carried out with DY-405 to Pfs230C1 molar ratio of 5:1. 100 µg of Pfs230C1 was dialysis into 100 mM sodium bicarbonate buffer (pH 9) for 4–6 h at 4 °C twice, and then labelled with DY-405 at room temperature for 1 h. Free dyes was removed by dialysis against PBS three times at 4 °C. Pfs25 was labelled with DY-490-NHS-Ester (DY-490, Dyomics) in a similar manner. Labelling was carried out with DY-490 to Pfs25 at molar ratio of 5:1. 100 µg of antigen was dialyzed into 100 mM sodium bicarbonate buffer (pH 9) for 4–6 h at 4 °C twice. And was later labelled with DY-490 at room temperature for 1 h, followed by dialysis against PBS three times at 4 °C to remove free dye.

### Fluorescent quenching assay

Fluorophore-labelled Pfs25 and Pfs230C1 were incubating with liposomes with a 1:4 mass ratio of total protein: CoPoP or PoP at a final antigen concentration at 40 μg/mL. The quenching of each samples was checked at 0.5, 1, 2, and 3 h at room temperature. To check the fluorescence signal, each of the incubation samples were diluted 200 times in PBS in a 96-well plate, and fluorescence was measured at excitation/emission at 491/515 nm for DY-490 labelled Pfs25 and excitation/emission at 400/420 nm for DY-405 labelled Pfs230. The percentage of binding was calculated based on the following formula:  % antigen binding = (1 − FL liposomes + antigen/FL antigen) × 100%, where FL stands for fluorescent intensity.

### Serum stability

The mixture of DY-490 labelled Pfs25 (80 µg/mL) and DY-405 labelled Pfs230C1 were incubated with CoPoP liposomes (320 µg/mL CoPoP, using the PHAD504 formulation) for 3 h at room temperature. An equal volume of 40% human serum, diluted in PBS was added to achieve a final concentration at 20% human serum. Samples were incubated at 37 °C for 0, 3, 7 and 14 days.

### Cryo-electron microscopy

10 µL of Pfs230C1 (80 µg/mL) and 10 µl of Pfs25 (80 µg/mL) were mixed with 20 µl of CoPoP liposomes with PHAD504 (320 µg/mL CoPoP) in PBS. Holey carbon grids (c-flat CF-2/2-2C-T) were washed with chloroform and glow discharged at 5 mA for 15 s immediately before the application of the sample. A volume of 3.6 µL of sample was deposited in the grid and vitrification was performed in a Vitrobot (ThermoFisher) by blotting the grids once for 3 s and blot force +2 before they were plunged into liquid ethane. Temperature and relative humidity during the vitrification process were maintained at 25 °C and 100%, respectively. Grids were loaded into a Tecnai F20 electron microscope operated at 200 kV using a Gatan 626 single tilt cryo-holder. Images were collected in a Gatan Ultrascan 4000 4 k × 4 k CCD Camera System Model 895 at a nominal magnification 60,000 × , which produced images with a calibrated pixel size of 1.8 Å/pixel. Images were collected with a total dose of ~ 50 e^−^/Å^2^ using a defocus ranging from − 2.7 to − 3.5 μm. Images were cropped and prepared for figures using Adobe Photoshop program.

### Murine immunization, serum and antibody analysis

5-week-old female CD-1 mice received intramuscular injections on days 0 and 21 containing the indicated antigens combined with CoPoP/MPLA liposomes with the following formulation, DPPC:CHOL:PHAD:CoPoP] [4:2:1:1]. The dose of antigens used is indicated in the corresponding figures; for the dual antigen immunization, 5 ng of total antigen contained 2.5 ng of Pfs25 and 2.5 ng of Pfs230C1; 25 ng of total antigen contained 12.5 ng of Pfs25 and 12.5 ng of Pfs230C1; and 50 ng of total antigen contained 25 ng of Pfs25 and 25 ng of Pfs230C1. Serum was collected on day 42 unless otherwise indicated and sent to the Laboratory of Malaria and Vector Research at the National Institute of Allergy and Infectious Diseases (NIAID) for anti-Pfs25 and anti-Pfs230C1 ELISA [26] and SMFA [27, 28] analysis which was carried out as previously described. In brief, the absorbance of each test sample (either serum or purified total IgG) was converted into ELISA units using a standard curve generated by serially diluting the standard (a pool of mouse anti-Pfs25 or anti-Pfs230 antisera) in the same ELISA plate. The ELISA unit value of a standard was assigned as the reciprocal of the dilution giving an O.D. _405_ = 1 in a standardized assay. The SMFA was conducted with 16–18 day old gametocyte cultures of the *Plasmodium falciparum* NF54 line, and female *Anopheles stephensi* mosquitoes were fed mixtures of gametocytes and test (or control) purified total IgGs at indicated concentrations through a membrane-feeding apparatus. All feeding experiments were performed with human complement (i.e., using non-heat-inactivated human serum). The blood-fed mosquitoes were kept for 8 days and dissected (n = 20 per group) to enumerate the oocysts in the midgut.

### New Zealand white rabbit immunization

10–12 weeks old Female rabbits received intramuscular injections on days 0 and 28 of 20 µg of Pfs230C1, Pfs25 or Duplex (10 µg Pfs25 plus 10 µg Pfs230C1) with CoPoP/MPLA504 liposomes, prepared by Monophosphoryl Lipid A-504 (PHAD504, Avanti # 699810) with a [DPPC: CHOL: PHAD504: CoPoP = 4: 2: 0.4: 1] mass ratio. Sera were collected on day 0, 28 and day 56. Serum was transferred to NIAID for ELISA and SMFA analysis.

### Indirect immunofluorescence assay (IFA)

*Plasmodium falciparum* gametocytes were obtained from the Johns Hopkins Parasitology Core Facility and fixed on slides as described before [19]. Slides were blocked with 5% BSA/PBS-T for 30 min at 37 °C. Sera collected from mice immunized with Pfs230C1, Pfs25 or the duplex, adjuvanted with CoPoP liposomes, was diluted 1:200 and incubated in 5% BSA/PBS with the fixed slides at 37 °C for 1 h, followed by 3 times washing with PBS in a humidity chamber, each time for 5 min. FITC-conjugated goat anti-mouse IgG (1:1000) was then incubated with the slides for 30 min at 37 °C, followed by 3 times washing with PBS, each time for 5 min. The slides were mounted with Prolong gold antifade with DAPI (# P36931) and imaged with an EVOS FL microscope using a 100× objective lens.

### Statistics

The 95% confidence interval and *p* value for TRA were calculated using a zero-inflated negative binomial model as previously described [29]. GraphPad Prism was used for statistical comparison of log-transformed ELISA data with one-way ANOVA followed by Tukey tests.

## Results

### Pfs230C1 and Pfs25 spontaneously bind to CoPoP/MPLA liposomes

**Fig. 1:**
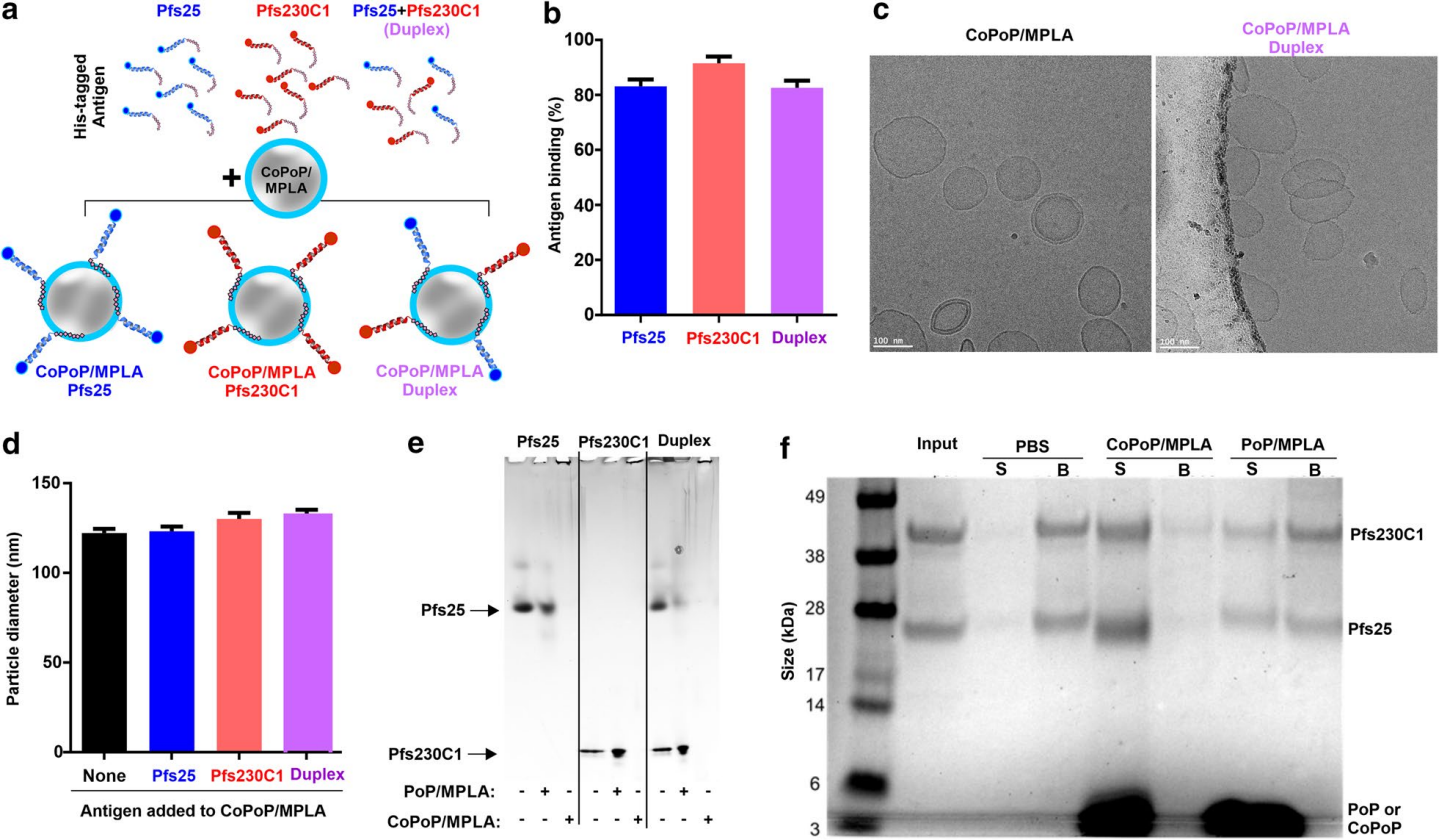
Duplexed particle formation of Pfs25 and Pfs230C1 with CoPoP/MPLA liposomes. **a** Schematic representation of the duplexing concept with CoPoP liposomes. **b** Binding of individual or duplexed antigens to CoPoP/MPLA liposomes following 3 h incubation, using a microcentrifugal filtration assay. **c** Cryo EM of CoPoP/MPLA liposomes before (left) and after binding duplexed Pfs25 and Pfs230 (right). **d** Particle size before and after antigen binding. **e** Native PAGE confirming individual and duplexed antigen binding to CoPoP/MPLA liposomes. The absence of a band in the CoPoP lane is because the antigens attached to the liposomes are too large to enter the gel. Note that Pfs230C1 runs faster than Pfs25 in native PAGE conditions. **f** SDS-PAGE showing whether the duplex antigens bind to Ni–NTA beads (“B”) or remain in the supernatant (“S”). The antigens bound to CoPoP liposomes remain in the supernatant since the his-tag stably anchors them into the bilayer without capacity to get captured by the beads. Bar graphs in **b** and **d** show mean ± std. dev. for n = 3 triplicate experiments

As shown in Fig. 1a the concept of duplexing in this study is to co-bind Pfs25 and Pfs230C1 onto liposomes by mixing the two antigens together with CoPoP liposomes. Liposomes were mixed with the individual antigens or the duplexed antigens for 3 h at room temperature at a 4:1 mass ratio of CoPoP:protein. The amount of antigen bound to the liposomes was then measured by a microcentrifugal filtration binding assay. Without binding to liposomes, 85% of the antigens could pass through the membrane and be recovered in the filtrate (Additional file 1: Fig. S1). After incubating with CoPoP liposomes, approximately 80% of the total protein bound to the liposomes for both the individual antigens as well as the duplex (Fig. 1b). The vaccine was used without any additional purification. Although the inter-particle distribution of the two antigens on liposomes in (the case of the duplex) was not characterized, since the antigens each bound greater than 80% when incubated with CoPoP liposomes individually or simultaneously, it is assumed that the antigens distributed together to a substantial extent. Cryo-electron microscopy with CoPoP/MPLA liposomes bearing duplexed antigens showed particles had a slightly oblong shape compared to control CoPoP/MPLA liposomes, which were spherical (Fig. 1c). This may be due to biophysical stresses induced on bilayer structure by the bound proteins. During the particleization process, the average size of the liposomes increased slightly after the incubation with Pfs25, Pfs230C1 or the duplex, without significant difference (Fig. 1d). The small size increase indicated that no major liposome aggregation was induced by antigen particleization, and there was no obvious change of the size distribution of CoPoP liposomes after incubated with the antigens (Additional file 1: Fig. S2). Native gel electrophoresis was used to directly confirm binding of both antigens, in monovalent and bivalent form, to CoPoP/MPLA liposomes, as indicated by the lack of a protein band when incubated with CoPoP liposomes, since the proteins were too large to enter the gel (Fig. 1e). The proteins did not bind to liposomes that lacked cobalt, but were otherwise identical (PoP/MPLA) and thus entered the gel. Note that in these native and non-denaturing conditions, Pfs230C1 migrates further than Pfs25, despite being nearly twice as large, likely due to the charge of the protein in native gel conditions.

To further ascertain particle formation and stability of the duplex vaccine, a denaturing SDS-PAGE assay was used. After mixing with CoPoP or PoP liposomes, the proteins were then mixed with Ni–NTA beads. These beads capture free his-tagged protein, whereas the liposome-bound protein does not get retained with the beads (“B”), but stays in the supernatant (“S”) suggesting stable and almost full binding of the proteins to the CoPoP in the liposomal bilayer. Figure 1F shows that the duplexed antigens with CoPoP did not bind to the Ni–NTA beads, whereas the free protein in PBS did. When mixed with PoP liposomes, most of the protein could be captured by the Ni–NTA beads. A minor fraction of protein appeared to remain in the supernatant, possibly due to adsorption to the PoP liposomes, or interference with the liposomes with the Ni–NTA binding.

**Fig. 2:**
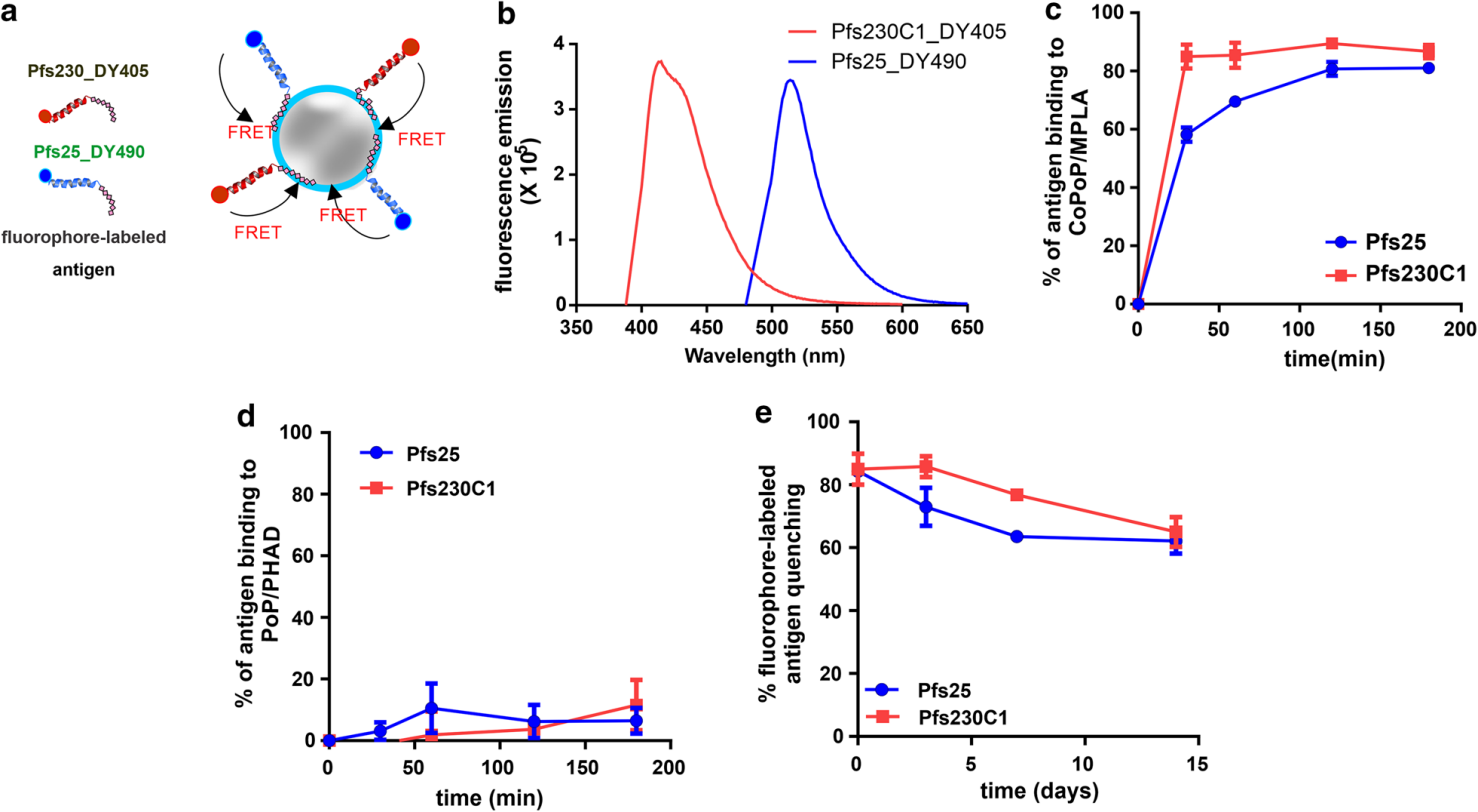
Pfs25 and Pfs230C1 binding to CoPoP/MPLA liposomes characterized using a FRET assay. **a** Schematic representation of fluorophore-labelled Pfs25 and Pfs230C1 which are quenched after binding to CoPoP/MPLA liposomes, **b** fluorescence emission spectra of fluorophore-labelled Pfs25 (blue) and Pfs230C1 (red). Based on fluorescence quenching, binding kinetics of the duplexed antigens was assessed in real-time with **c** CoPoP/MPLA liposomes and **d** PoP/MPLA liposomes, which lack cobalt in the bilayer. **e** Serum stability of the duplexed antigens with CoPoP/MPLA liposomes during incubation for 2 weeks in 20% human serum at 37 °C. Data in **c**, **d**, and **e** show mean ± std. dev. for n = 3 triplicate experiments

Next, a fluorescence resonance energy transfer (FRET) assay was developed to assess binding kinetics and stability for the individual antigens of the duplexed vaccine (Fig. 2a). Pfs230C1 and Pfs25 were covalently labelled with small molecule fluorophores of different colors in order to track fluorescent quenching, which occurs upon binding to the liposomes, due to energy transfer to the porphyrin chromophore. Fluorophore-labelled Pf230C1 and fluorophore-labelled Pf25 had minimal overall spectral overlap in emission, as shown in Fig. 2b. The quenching of Pfs25 and Pfs230C1 both reached approximately 80% after 3 h incubation with CoPoP/MPLA liposomes (Fig. 2c) but not with PoP/MPLA liposomes (Fig. 2d). Pfs230C1 bound to liposomes somewhat more quickly than Pfs25 for reasons that are not clear. It is possible that the his-tag is more accessible in the case of Pfs230C1 to more rapidly bind with the cobalt in the bilayer. To assess serum stability assay, liposomes bearing the fluorescent antigens were incubated with 20% human serum at 37 °C for 2 weeks, and quenching was assessed on day 0, 3, 7 and 14 (Fig. 2e). Both antigens remained quenched during this long serum incubation, albeit with some decrease over time. This suggests that the majority of antigens remain stably bound to the liposomes in physiological conditions.

### Immunization with bivalent particles induces functional IgG in mice

It has previously been demonstrated that Pfs25 and Pfs230, individually, induce functional IgG when particleized with CoPoP liposomes [18, 19]. To investigate whether a bivalent particle vaccine could generate antibodies against the individual antigens, outbred female mice were immunized with a fixed total mass dose of antigen with CoPoP/MPLA liposomes with 50 or 5 ng of total antigen dose. The antigens and liposomes were not purified after mixing. The total antigen dose was kept constant to facilitate assessing whether antibody function is improved in the duplexed vaccines relative to individual ones, since both antigens induce the same type of transmission-blocking function.

**Fig. 3:**
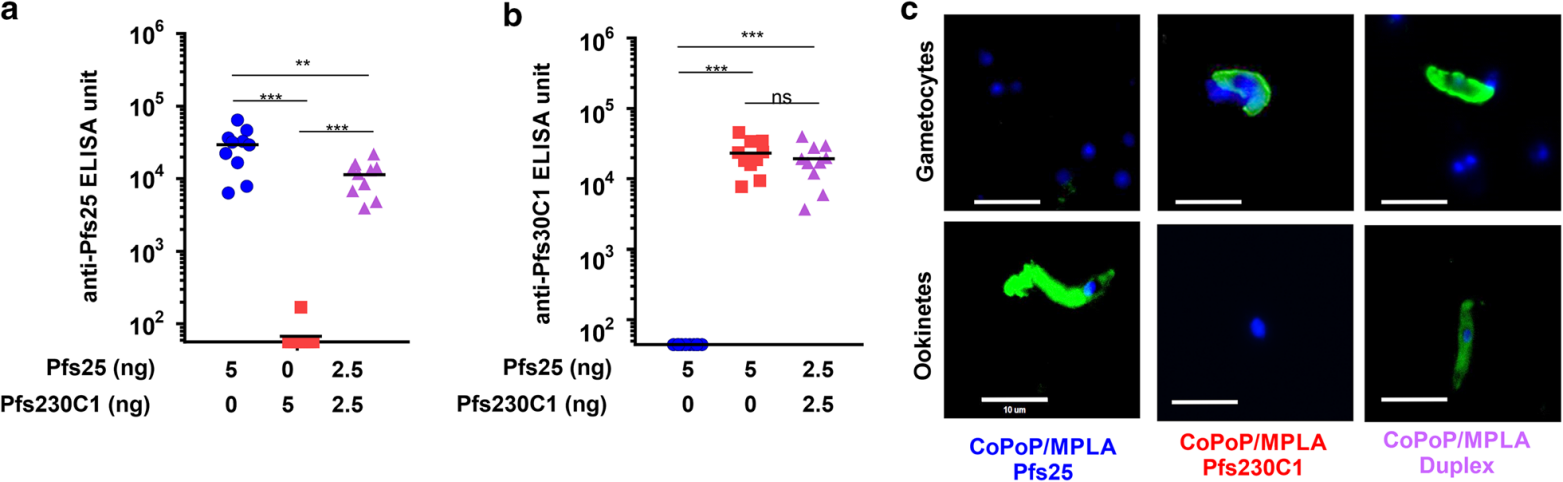
Specificity of antibodies induced by mice immunized with duplexed or individual antigens with CoPoP/MPLA liposomes. CD-1 mice were immunized intramuscularly with Pfs25, Pfs230 or the bivalent combination, admixed with CoPoP/MPLA liposomes on day 0 and day 21. Sera were collected on day 42. **a** Anti-Pfs25 and **b** Anti Pfs230C1 IgG titres were assessed. ELISA experiments were performed with n = 10 mice and the line represents the geometric mean. One-way ANOVA followed by Tukey test, using log transformed data was used to compared difference^. **^p = 0.0058, ^***^p < 0.0001. **c** Indirect immunofluorescence assay of NF54 parasites at gametocyte and ookinete stages, reacted with indicated post-immune mouse sera. Images were taken from a single experiment

Mice were immunized on day 0, boosted on day 21, and sera were collected for evaluation on day 42. When 50 ng of total antigen was used for immunization, all immunization groups (i.e. Pfs25, Pfs230C1 and the duplex) yielded specific antibodies against Pfs25 and Pfs230C1 (Additional file 1: Fig S3A) that exhibited strong TRA (Additional file 1: Fig S3B), making comparison between groups difficult. Therefore, since groups immunized with 50 ng of antigens showed no difference in SMFA, sera were assessed from mice immunized with 5 ng of total antigen using SMFA at decreasing IgG concentration. The 5 ng protein dose was adjuvanted with CoPoP liposomes (20 ng CoPoP and 20 ng MPLA). Following prime and boost immunization, the duplex generated specific antibodies against both Pfs25 (Fig. 3a) and Pfs230C1 (Fig. 3b). As expected, immunization with individual antigens yielded only the antibodies specific for the immunization antigen. Immunization with 5 ng Pfs25 alone induced significantly more anti Pfs25 IgG compared to the duplex, which also had 5 ng of total antigen, but just half the Pfs25 dose (2.5 ng). The geometric mean for the Pfs25 ELISA units induced by the single antigen was twice that compared to the duplexed antigen (geometric mean of 23,900 *vs* 10,000). In contrast, immunization with Pfs230 induced only slightly higher levels of Pfs230 ELISA units compared to the duplex, and the difference was not statistically significant (geometric mean 23,100 vs 19,100). Thus, for the Pfs25, reducing the antigen dose in half and duplexing it with Pfs230C1 resulted in a reduction in the induced antibodies by approximately half. But for Pfs230C1, reducing the antigen dose in half and duplexing it with Pfs25 did not significantly decrease the obtained anti-Pfs230C1 antibodies. Similar results were observed not just with immunization at a 5 ng total antigen dose, but also at a 25 ng total antigen dose and a 50 ng total antigen dose (Additional file 1: Tables S1 and S2). At the 50 ng total dose, mice immunized with the single antigen Pfs230C1 elicited slightly cross-reactive antibodies to Pfs25 (geometric mean 151), but the level of anti-Pfs25 antibodies was much lower than either the Pfs25 alone or the duplex groups (geometric mean of 41,613 and 21,484, respectively).

Antibody reactivity with gametocytes and ookinetes was assessed using an immunofluorescence assay (IFA) (Fig. 3c). Antibodies generated from mice immunized with particleized Pfs230C1 in individual or duplexed form recognized Pfs230 proteins on the surface of gametocytes. Likewise, immunization with Pfs25 and the duplex induced antibodies that reacted with Pfs25 on the surface of ookinetes. Therefore, immunization with CoPoP/MPLA liposomes induced antibodies which recognized the native protein on the parasite surface regardless of whether antigens were duplexed or not.

**Fig. 4:**
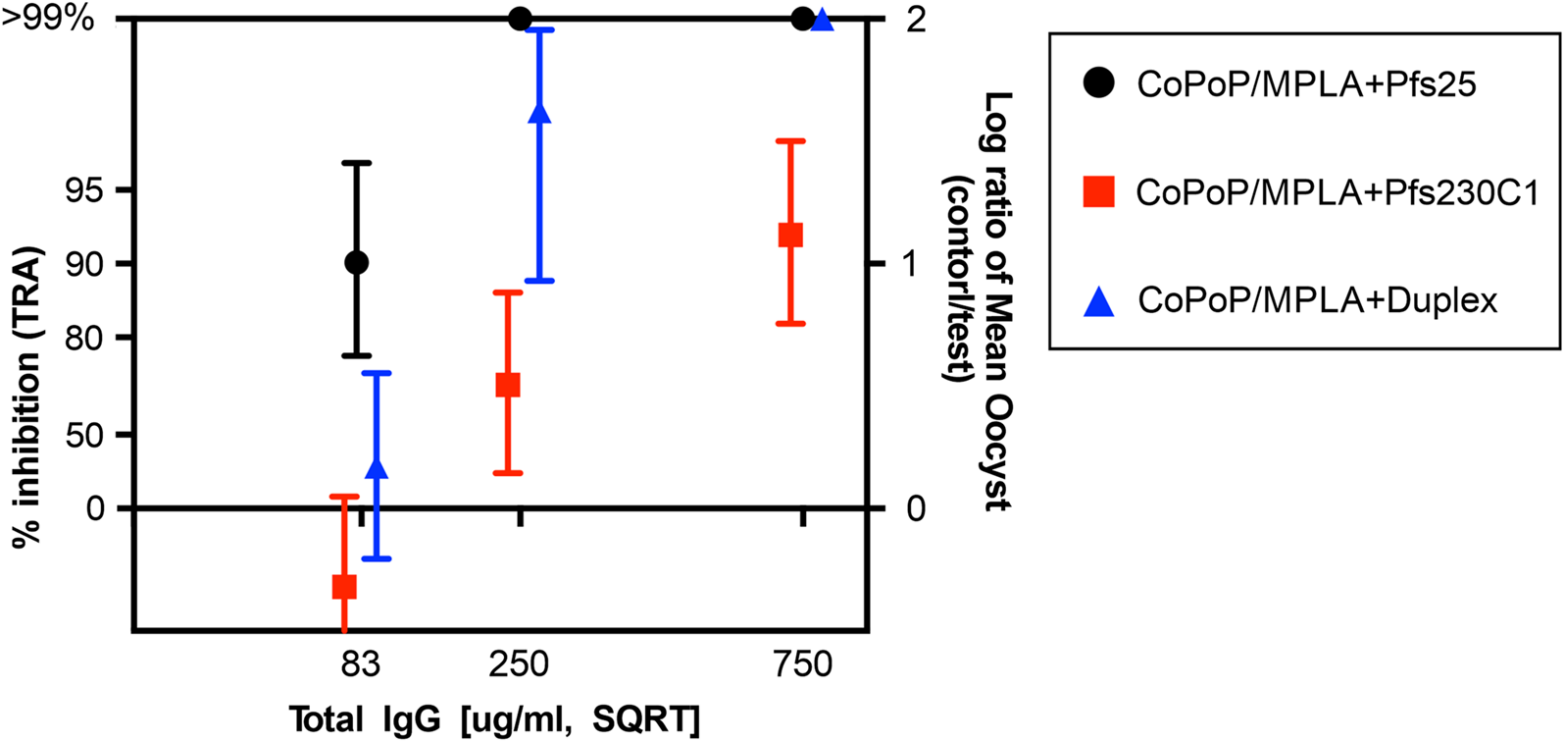
Duplex immunization with CoPoP/MPLA liposomes does not induce mouse antibodies with improved function. Mice were immunized with 5 ng total protein (individual or duplexed) and IgG was purified form pooled sera (n = 10 mice per group). Purified IgG were fed to n = 20 mosquitoes per group at the indicated IgG in a concentrations in the standard membrane feeding assay (SMFA) and oocysts were counted 8 days later. Transmission-reducing activity corresponds to the reduction of oocyst number compared to the adjuvant alone control. The error bar indicates 95% confidence intervals

Purified IgGs from pooled serum from mice immunized with the individual or duplexed antigens was assessed for functional activity in the SMFA (Fig. 4, Additional file 1: Tables S3, S4 and S5). More than 92% inhibition in oocyst density was observed with 750 μg/mL purified IgG for all Pfs25, Pfs230 or the duplex. In order to observe a difference among the antibodies, the feeding assay was repeated using lower IgG concentrations of 250 and 83.3 μg/mL. This range of IgG concentration revealed a dose-dependent response, with lower antibody concentrations yielding reduced TRA for the Pfs230 and duplex post-immune sera. The post-immune sera from immunization with just 5 ng Pfs25 exhibited strong TRA at 83 ug/mL IgG in the SMFA.

### Immunization with bivalent particles induces functional IgG in rabbits

**Fig. 5:**
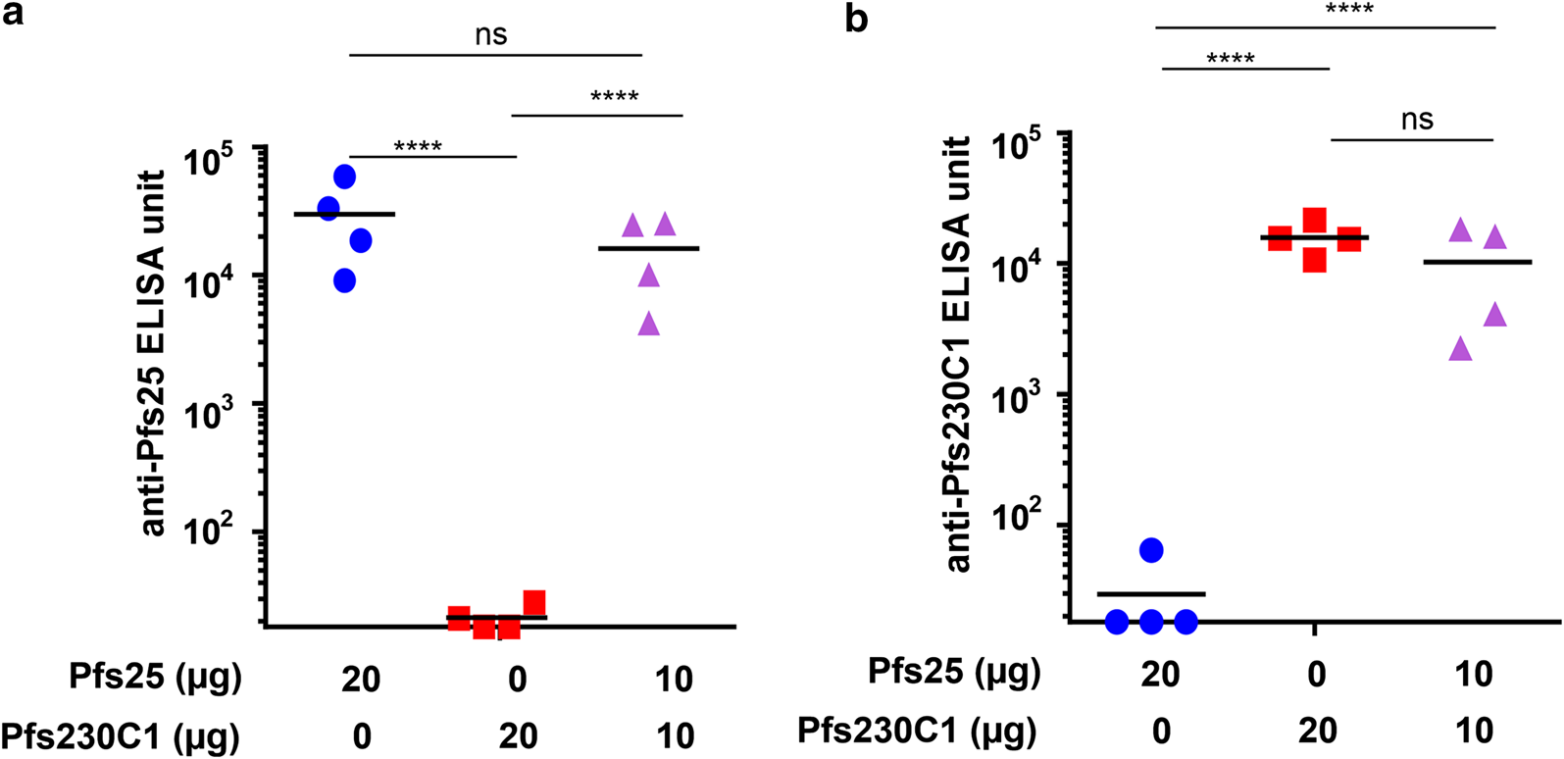
Immunization of rabbits with Pfs25 and Pfs230C1 elicits specific antibodies using duplexed and individual antigens with CoPoP liposomes. Rabbits were immunized with 20 μg of total Pfs25, Pfs230 or duplexed antigens with CoPoP/MPLA liposomes on day 0 and day 28, and final bleeding was collected on day 56. **a** Pfs25 and **b** Pfs230C1 ELISA IgG titre. ELISA experiments were performed with n = 4 of independent rabbits and line represent geometric mean. One-way ANOVA followed by Tukey test using log transformed data was used to compare difference^. ****^p < 0.0001

**Fig. 6:**
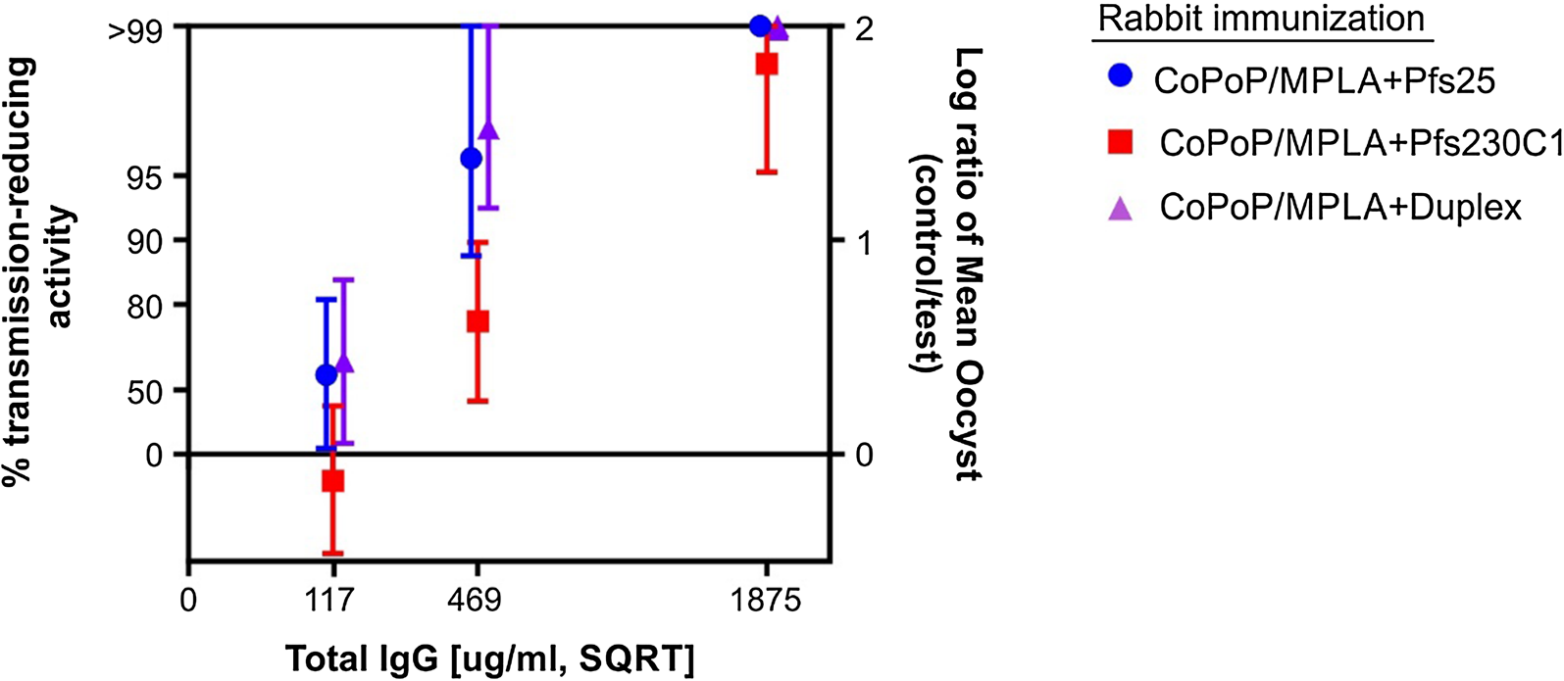
Duplex immunization with CoPoP liposomes does not induce rabbit antibodies with improved function compared to Pfs25 alone. Rabbits were immunized with 20 µg total antigen on day 0 and day 28, then day 58 IgG was purified from pooled sera (n = 4 rabbits per group). The SMFA was carried at indicated IgG concentrations. The error bars indicate 95% confidence intervals

Duplex immunization was next assessed in rabbits, by intramuscular immunization with 20 μg of total antigen; particleized Pfs230C1, Pfs25 or the duplex (10 μg of Pfs230C1 and 10 μg of Pfs25). This antigen dose level is similar to that which was previously shown to be effective in rabbits with Pfs25 [19]. Rabbits were immunized with a modified liposome formulation that used structurally-similar PHAD504 instead of PHAD. This formulation was found to particleize Pfs25 and Pfs230 and maintain a size of around 100 nm (Additional file 1: Fig. S4) similarly to the PHAD formulation. Rabbits were primed on day 0, boosted on day 28, and day 56 sera were collected and assessed. The post-immune serum showed the presence of anti-Pfs25 IgG (Fig. 5a) and anti-Pfs230C1 IgG (Fig. 5bb) on day 56. The duplex did not yield significantly higher or lower levels of individual antibodies compared to the individual antigen immunization. When the purified IgG from individual rabbits was tested in SMFA at 3750 μg/mL IgG, all showed 99–100% inhibition (Additional file 1: Table S6). Pooled IgG was prepared for each group for further testing at lower antibody concentrations. When purified IgGs from the pooled, post-immune rabbit sera were assessed in SMFA at 1875 μg/mL IgG concentration, nearly complete TRA was again observed for all immunization groups (Fig. 6, Additional file 1: Tables S6 and S7). However, at lower IgG concentrations of 469 μg/mL, TRA was diminished for the Pfs230C1 post-immune sera, whereas the duplex and the Pfs25 post-immune sera exhibited close to full TRA. At an IgG concentration of 117 μ g/mL, TRA was reduced for all post-immune sera, with the same trend of Pfs230C1 inducing the least inhibition of parasite transmission, and the duplex and Pfs25 post-immune sera providing comparable activity.

## Discussion

CoPoP liposomes readily produced bivalent particles decorated with Pfs230C1 and Pfs25. Multiple biochemical assays based confirmed that the antigens bind to CoPoP liposomes, but not PoP liposomes. A fluorometric assay using spectrally-distinct fluorophore labelling of each antigen showed that they could rapidly bind the liposomes simultaneously with serum stability. Overall, immunization with the antigens proved to be potent, with strong functional antibody activity observed with individual or duplexed antigens in mice immunized with just a 5 ng dose (along with 20 ng MPLA and 20 ng CoPoP). This is consistent with previous observations of the potency of this adjuvant system, which was attributed at least in part to enhanced uptake of the antigen in particle form into immune cells in draining lymph nodes [19].

Immune interference effects should be evaluated when combining two antigens. The interference could change not only quantity of induced antibody, but also quality. In the present study, in mice, compared to single antigen immunization, Pfs230C1 antibody levels were not diminished in the duplex vaccine, but the Pfs25 antibody levels were reduced approximately in half. The antigen dose for individual antigen immunization was twice that of the duplex, which might contribute to this. Experiments were not designed to assess interference, however post hoc analysis of groups immunized with 25 ng Pfs25 with or without addition of 25 ng of Pfs230 showed there was a significant reduction in the Pfs25 antibody levels, when comparing log-transformed ELISA units (one-tailed unpaired T-test, p = 0.02; Additional file 1: Table S1). Conversely, when mice were immunized with 25 ng Pfs230C1, no significant difference in anti-Pfs230C1 antibody levels was observed with or without the presence of 25 ng Pfs25 on the particles (one-tailed unpaired T-test, p = 0.21; Additional file 1: Table S2). In rabbits, no significant decreases in antibody yield were observed for the duplex compared to the individual antigens (Fig. 5), although duplex group received half the dose of the single antigens compared to the single antigen groups. The reasons for this discrepancy between rabbits and mice is not clear, but might be explained, at least in part, by the doses of protein used. For the mouse study, nanograms of Pfs25 protein were compared (which might be in the middle part of a dose–response), while 20 and 10 micrograms were used in rabbits (which could be towards a plateau of dose–response). Another possibility is that the number of rabbits used was insufficient (n = 4 in the rabbit study, while n = 10 for mouse study) to reach statistical significance. Antibody quality (judged by transmission-reducing activity at the same total IgG concentrations) of duplexed groups was better than that in single-antigen Pfs230 immunization both in mice and rabbits, however, it was equally good (Fig. 6 in rabbits) or worse (Fig. 4 in mice) than Pfs25 single immunization groups, when the same total antigen doses were administered. Addition of Pfs25 to an HIV-derived peptide using CoPoP liposomes was reported to induce higher antibody titres against the peptide, although those antibodies had diminished function [30]. This may have resulted from Pfs25 providing additional helper T cell epitopes that enhance the immunogenicity of the HIV peptide, but at the same time the protein might interfere with the peptide conformation, resulting in less functional antibodies. Compared to that study, any changes in Pfs25 and Pfs30 antibody response induced by duplexing, compared to monovalent immunization, appeared modest. Among single immunization groups, Pfs25 groups showed higher inhibitions than Pfs230 groups both in mice and rabbits. The mechanism of difference had not been investigated in this study, and it could be a combination of many complicated factors (e.g., the Pfs25 vaccine induced more antigen-specific antibodies in a mass concentration, the Pfs25 vaccine induced polyclonal antibodies which recognized more functional epitopes than non-functional epitopes). Further studies are required to better determine immuno-dominant effects of Pfs230 on the Pfs25 antibody response, and the mechanism of difference in SMFA activity between the two antigens.

The results presented here did not show, under any conditions, that the duplex vaccine enhanced the TRA of the induced antibodies compared to immunization with an equivalent total protein dose of Pfs25. These results are generally consistent with a recent study that examined a Pfs25 (aa 22-193)-Pfs230C (aa443-1132) fusion and did not find an advantageous effect compared to single antigens [20]. It should be noted however that another study recently did report evidence of enhancement in developing of a Pfs230 and Pfs48/45 fusion antigen [31]. It is not clear whether different types of transmission-blocking antigen targets could be better suited for duplexing than Pfs25 and Pfs230. However, using distinct antigens which target independent life stages of the parasite (i.e. mosquito, pre-erythrocytic or blood-stage), so induced antibodies have distinct functional activities, would have advantages of more interpretable analysis of functional activity.

## Conclusions

Multiplexing recombinant antigens is an intriguing approach for malaria vaccine development and can be carried out readily using CoPoP liposomes. This study found that CoPoP liposomes potently induced functional antibodies using two individual or duplexed TBV antigens in mice and rabbits. However, compared to using a single, effective transmission blocking antigen, no advantages were observed for duplexing Pfs25 and Pfs230, which target ookinetes and gametes in the mosquito stage of the parasite, respectively. Duplexing of a mosquito-stage antigen with another life stage antigen may be a useful future direction to consider.

## **Supplementary information**

**Additional file 1.** Additional Figures S1–S4 and Tables S1–S7.

## Data Availability

All raw data are available upon request.

## Abbreviations

CoPoP: Cobalt-porphyrin-phospholipid
CHOL: Cholesterol
DPPC: 1,2-dipalmitoyl-sn-glycero-3-phosphocholine
FRET: Fluorescence resonance energy transfer
CSP: Circumsporozoite protein
MPLA: Monophosphoryl lipid A
PBS: Phosphate buffered saline
PHAD: Phosphorylated HexaAcyl Disaccharide
PoP: Porphyrin-phospholipid
TBV: Transmission-blocking vaccine
TRA: Transmission reducing activity
