# Supplementary material for: Particle-based, Pfs230 and Pfs25 immunization is effective, but not improved by duplexing at fixed total antigen dose
Source: Malar J. 2020 Aug 28;19:309. doi: 10.1186/s12936-020-03368-5 (PMC7453371; doi:10.1186/s12936-020-03368-5)
Supplement: Supplementary file 1 — Additional file 1. Additional Figures S1–S4 and Tables S1–S7. [file 12936_2020_3368_MOESM1_ESM.pdf]

Supplementary Data

Particle-based, Pfs230 and Pfs25 immunization is effective, but not improved by duplexing at fixed total antigen dose

Wei-Chiao Huang[1], Bingbing Deng[2], Moustafa T Mabrouk[1], Amal Seffouh[3], Joaquin Ortega[3], Carole Long[2], Kazutoyo Miura[2], Yimin Wu[4], Jonathan F. Lovell[1]

[1]Department of Biomedical Engineering, University at Buffalo, State University of New York, Buffalo, New York 14260, USA

[2]Laboratory of Malaria and Vector Research, National Institute of Allergy and Infectious Diseases, National Institutes of Health, Rockville, Maryland 20852, USA

[3]Department of Anatomy and Cell Biology, McGill University Montreal, Quebec, H3A 0C7, Canada

[4]PATH's Malaria Vaccine Initiative (MVI), Washington, DC 20001, USA

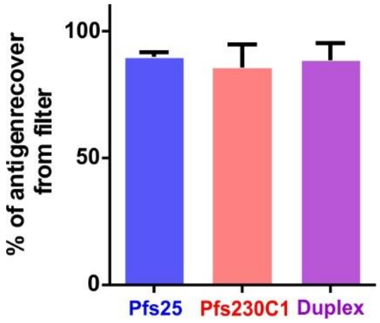

Additional File: Fig S1. Micro BCA were used to measured antigen concentration before and after samples were pass through the microcentrifugal filtration. The bar graph represent mean +/- std. dev. for n=3 experiments.

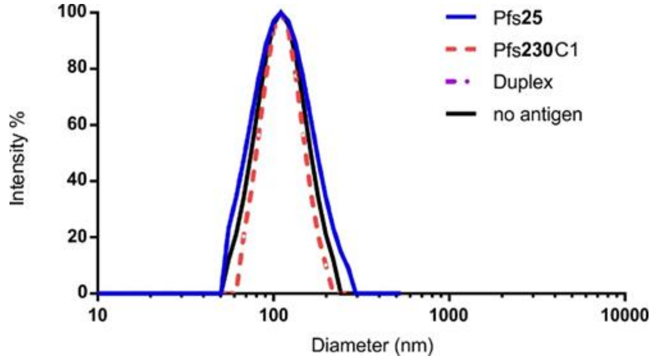

Additional File: Fig S2. Liposome size distribution of CoPoP/MPLA liposomes measured by DLS.

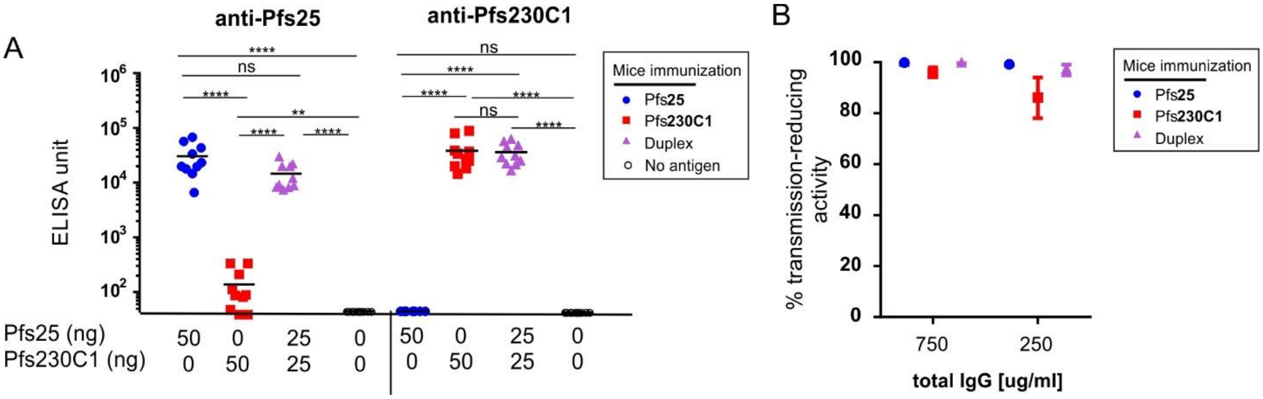

Additional File: Fig S3. Mice immunized with CoPoP/MPLA liposomes generated functional antibodies with strong transmission-reducing activity at 50 ng total antigen dose. Mice were immunized with 50 ng of Pfs25, 50 ng of Pfs230C1 or 25 ng Pfs25 plus 25 ng Pfs230C1 with CoPoP/MPLA liposomes on day 0 and day 21, final bleeding were collected on day 42. (A) Anti-Pfs25 and anti-Pfs230C1 ELISA titer. (B) SMFA activity were assessed at indicated concentration of purified IgG from serum. One-way ANOVA followed by a post-hoc Tukey's test, $^{**}p<0.01$, $^{****}p<0.0001$. Error bars indicate 95 % confidence intervals.

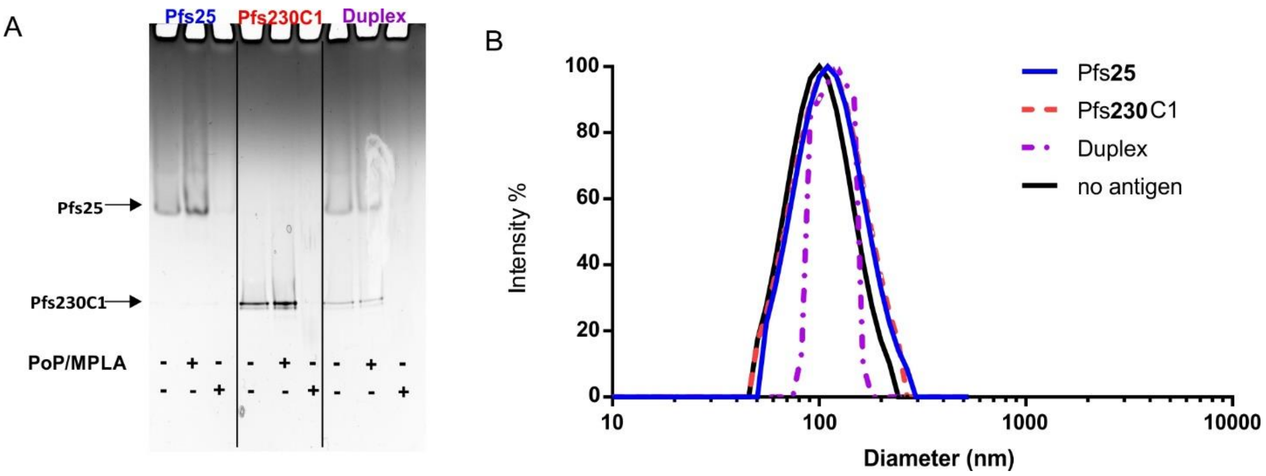

Additional File: Fig S4. CoPoP/PHAD504 liposomes particleize duplexed antigens. A) Native PAGE confirms single and duplexed antigen binding to CoPoP/PHAD504 liposomes. **B)** Size distribution following antigen binding. These liposomes were used for rabbit studies.

Additional File: Table S1. Anti Pfs25 response following mouse immunization with individual or duplexed Pfs25 and Pfs230 at a constant antigen dose.

	Gr 1	Gr 2	Gr 3	Gr 4	Gr 5	Gr 6	Gr 7	Gr 8	Gr 9	Gr 10
**Ag**	Pfs25	Pfs230	Pfs25+ Pfs230	Pfs25	Pfs230	Pfs25+ Pfs230	Pfs25	Pfs230	Pfs25 +Pfs230	No antigen
**Adjuvant**	CoPoP/MPLA									
**dose [ng]**	5	5	2.5+2.5	50	50	25+25	25	25	12.5+12.5	0.0
AN# 1	22,283	56	14,018	33,077	507	15,140	19,639	56	16,748	56
AN# 2	46,255	56	11,687	24,314	166	50,849	33,008	56	34,365	56
AN# 3	36,430	56	13,054	32,797	129	37,194	22,338	56	4,996	56
AN# 4	64,212	56	6,779	116,061	69	19,994	40,420	56	33,653	56
AN# 5	32,606	56	8,423	29,710	507	13,401	18,020	56	23,410	56
AN# 6	31,792	56	15,774	38,977	120	33,334	65,113	56	26,807	56
AN# 7	6,339	56	13,249	97,670	131	33,720	46,449	56	29,263	56
AN# 8	29,191	56	4,815	10,761	56	14,615	199,187	56	28,557	56
AN# 9	7,827	56	21,689	56,752	56	13,656	38,272	56	23,609	56
AN# 10	16,534	169	3,910	73,628	320	12,173	37,147	56	3,078	56
**GeoMean**	**23,862**	**63**	**10,041**	**41,613**	**151**	**21,484**	**39,408**	**56**	**18,058**	**56**

All ELISA data which were less than minimal detection level of assay are assigned as 56 in the table.

AN# refers to the animal number.

Additional File: Table S2. Anti Pfs230C1 response following mouse immunization with individual or duplexed Pfs25 and Pfs230C1 at a constant antigen dose.

	Gr 1	Gr 2	Gr 3	Gr 4	Gr 5	Gr 6	Gr 7	Gr 8	Gr 9	Gr 10
**Ag**	Pfs25	Pfs230	Pfs25+ Pfs230	Pfs25	Pfs230	Pfs25+ Pfs230	Pfs25	Pfs230	Pfs25+ Pfs230	No antigen
**adjuvant**	CP									
**dose [ng]**	5	5	2.5+2.5	50	50	25+25	25	25	12.5+12.5	0.0
AN# 1	44	34,122	3,668	44	37,055	44,459	78	8,313	23,527	44
AN# 2	44	15,916	5,905	44	33,817	63,101	44	22,092	35,415	44
AN# 3	44	7,708	27,738	44	27,209	57,750	44	120,382	10,399	44
AN# 4	44	23,605	11,993	44	80,077	16,720	44	39,009	43,611	44
AN# 5	44	23,973	16,783	44	14,345	29,203	44	4,224	26,484	44
AN# 6	44	9,345	39,799	44	18,218	22,476	68	22,906	14,097	44
AN# 7	44	18,168	19,908	44	19,974	48,106	44	18,353	29,438	44
AN# 8	44	18,641	29,830	44	25,146	25,295	44	74,317	33,566	44
AN# 9	44	34,559	19,208	44	38,632	31,593	44	52,765	32,114	44
AN# 10	44	45,390	17,113	44	87,966	21,586	44	12,650	21,809	44
**GeoMean**	**44**	**20,279**	**15,750**	**44**	**32,252**	**32,877**	**48**	**24,438**	**25,059**	**44**

All ELISA data which were less than minimal detection level of assay are assigned as 44 in the table.

Supplementary Tables of SMFA results

Each SMFA test group (i.e. each table entry) was assessed with n=20 mosquitos per group. Table entries indicate the sample dose used for immunization. Statistical testing is based on a zero inflated negative binomial random effects model that has been described previously (Miura et al. Vaccine. 2016;34:4145-51).

Additional File: Table S3. SMFA #239

	IgG conc. (ug/ml)	Average oocytes	% inhibition	p value
Gr10: CoPoP/MPLA alone	750	38.7	-	-
Gr 1: Pfs25 (5 ng) + CoPoP/MPLA	750	0	100	0.001
Gr 2: Pfs230 (5 ng) + CoPoP/MPLA	750	3.0	92.4	0.001
Gr 3: Pfs25 (2.5 ng) + Pfs230 (2.5 ng) + CoPoP/MPLA	750	0.1	99.9	0.001
Gr 4: Pfs25 (50 ng) + CoPoP/MPLA	750	0.1	99.9	0.001
Gr 5: Pfs230 (50 ng) + CoPoP/MPLA	750	1.8	95.5	0.001
Gr 6: Pfs25 (25 ng) + Pfs230 (25 ng) + CoPoP/MPLA	750	0	100	0.001

Additional File: Table S4. SMFA #247

	IgG conc. (ug/ml)	Average oocytes	% inhibition	p value
Gr10: CoPoP/MPLA alone	250	12.7	-	-
Gr 1: Pfs25 (5 ng) + CoPoP/MPLA	250	0	100	0.001
Gr 2: Pfs230 (5 ng) + CoPoP/MPLA	250	4.0	68.8	0.006
Gr 3: Pfs25 (2.5 ng) + Pfs230 (2.5 ng) + CoPoP/MPLA	250	0.3	97.6	0.001
Gr 4: Pfs25 (50 ng) + CoPoP/MPLA	250	0.1	99.2	0.001
Gr 5: Pfs230 (50 ng) + CoPoP/MPLA	250	1.8	86.2	0.001
Gr 6: Pfs25 (25 ng) + Pfs230 (25 ng) + CoPoP/MPLA	250	0.3	97.6	0.001

Additional File: Table S5. SMFA #254

	IgG conc. (ug/ml)	Average oocytes	% inhibition	p value
Gr10: CoPoP/MPLA alone	750	10.1		
Gr 1: Pfs25 (5 ng) + CoPoP/MPLA	83	1.0	90.1	0.001
Gr 2: Pfs230 (5 ng) + CoPoP/MPLA	83	21.1	-108.4	0.083
Gr 3: Pfs25 (2.5 ng) + Pfs230 (2.5 ng) + CoPoP/MPLA	83	6.8	32.7	0.367

Additional File: Table S6. SMFA #251

	IgG conc. (ug/ml)	Average oocytes	% inhibition	p value
Day 0 pooled IgG	3750	5.3	-	-
Rabbit1_20μg Pfs25	3750	0.0	100.0	0.001
Rabbit2_20μg Pfs25	3750	0.0	100.0	0.001
Rabbit3_20μg Pfs25	3750	0.0	100.0	0.001
Rabbit4_20μg Pfs25	3750	0.0	100.0	0.001
Rabbit1_20μg Pfs230C1	3750	0.1	99.1	0.001
Rabbit2_20μg Pfs230C1	3750	0.0	100.0	0.001
Rabbit3_20μg Pfs230C1	3750	0.1	99.1	0.001
Rabbit4_20μg Pfs230C1	3750	0.0	100.0	0.001
Rabbit1_20μg Duplex	3750	0.1	99.1	0.001
Rabbit2_20μg Duplex	3750	0.0	100.0	0.001
Rabbit3_20μg Duplex	3750	0.1	99.1	0.001
Rabbit4_20μg Duplex	3750	0.0	100.0	0.001

Additional File: Table S7. SMFA #262

	IgG conc. (ug/ml)	Average oocytes	% inhibition	p value
Day 0 pooled IgG	1,875	13.3	-	-
Rabbit Pool_20µg Pfs25	1,875	0.0	100.0	0.001
Rabbit Pool _20µg Pfs25	469	0.6	95.9	0.001
Rabbit Pool _20µg Pfs25	117	5.7	57.5	0.031
Rabbit Pool _20µg Pfs230C1	1,875	0.2	98.5	0.001
Rabbit Pool _20µg Pfs230C1	469	3.2	75.9	0.002
Rabbit Pool _20µg Pfs230C1	117	17.7	-32.7	0.473
RabbitPool_20µg Duplex	1,875	0.0	100.0	0.001
Rabbit Pool _20µg Duplex	469	0.4	97.0	0.001
Rabbit Pool _20µg Duplex	117	4.9	63.2	0.024
